# Technological Change in the Retirement Transition and the Implications for Cybersecurity Vulnerability in Older Adults

**DOI:** 10.3389/fpsyg.2020.00623

**Published:** 2020-04-30

**Authors:** Benjamin A. Morrison, Lynne Coventry, Pam Briggs

**Affiliations:** Psychology and Communication Technology Lab, Department of Psychology, Faculty of Health and Life Sciences, Northumbria University, Newcastle upon Tyne, United Kingdom

**Keywords:** retirement transition, cybersecurity, older adults, ageing, HCI

## Abstract

Retirement is a major life transition, which leads to substantial changes across almost all aspects of day-to-day life. Although this transition has previously been seen as *the* normative marker for entry into older adulthood, its influence on later life has remained relatively unstudied in terms of technology use and cybersecurity behaviours. This is problematic as older adults are at particular risk of becoming victims of cyber-crime. This study aimed to investigate which factors associated with the retirement transition were likely to increase vulnerability to cyber-attack in a sample of 12 United Kingdom based older adults, all of whom had retired within the past 5 years. Semi-structured, one to one interviews were conducted and subsequently analysed using thematic analysis. Six themes were identified referring to areas of loss in: social interaction, finances, day-to-day routine, feelings of competence, sense of purpose, and technology support structures. We discuss the implications of these losses for building cyber-resilience in retirees, with suggestions for future research.

## Introduction

Retirement is a major life transition in which nearly all aspects of life change (Salovaara et al., 2010) and has previously been seen as “*the* psychosocial marker for entry into old age” (Kloep and Hendry, 2006). The retirement transition offers both challenges and opportunities and can be seen as a period of loss, reconstruction, and renegotiation of varying aspects of life (Price, 2003; Salovaara et al., 2010; Mao et al., 2017). Some aspects of retirement, such as changes to one’s socio-economic environment and choosing how to spend newly acquired free time, have been consistent for generations of existing retirees. However, the rapid development and growth of technology provides a range of novel challenges and opportunities for those currently transitioning into retirement, and for those who will retire in the future. Technology may provide benefits to retiring adults, offering a solution to difficulties in navigating the transition to retirement. Conversely, technology may lead to additional challenges for those transitioning into retirement, such as an increased vulnerability to online victimisation.

The “Baby Boomer” generation – those born between 1946 and 1964 (Young and Tinker, 2017) – are currently making the transition to retirement and are likely to be the first generation to experience the costs and benefits of this technological change. This generation has lived through a digital revolution and are likely the first retirees to have used technology for a large part of their working lives (Durrant et al., 2017). Their engagement with technology makes them the first generation who are likely to use technology before, during and well into retirement. Technology use by this generation has steadily increased over time. For example, around 50% of this age group in the United Kingdom own a smartphone and those who say they never use the internet has dropped from 49 to 29% in the last 5 years (Ofcom, 2018).

Early research into technology use in older adults (for example: Gregor et al., 2002) was based on the premise that those in retirement were not active technology users. The concept and associated language, metaphors, and behaviours were unfamiliar to them, and they did not necessarily perceive the benefits of technology use. Today’s older adult population demonstrate a normative shift towards technology, with older adults eager to adopt new technology (Mitzner et al., 2010; Vaportzis et al., 2017), recognising its utility for maintaining independence for longer into older age (Lindley et al., 2008; Seifert and Schelling, 2018). Many older adults now engage with online technologies to counteract loneliness and isolation (Chopik, 2016), remain socially connected (Hutto and Bell, 2014), interact with family, and enjoy a healthier retirement (Khvorostianov et al., 2012; Juárez et al., 2018). However, these benefits are associated with known costs in terms of an increased risk of online victimisation for this population (Chakraborty et al., 2013; Sarno et al., 2017).

All digital technology users are potential victims of cyber-attacks and may even unknowingly participate in those attacks (Von Solms and Van Niekerk, 2013). Unsurprisingly, a growing body of cybersecurity research focusses on the role of the user. Here the drive is to understand the role of end-user behaviours and attitudes which range from authentication behaviours (Nicholson et al., 2013a, b), web browsing (Kisekka et al., 2015), decision making (Jeske et al., 2016), through the risky behaviours such as password sharing (Whitty et al., 2015).

Despite this growing literature base, there remains a paucity of cybersecurity research that explores the older adult user, even though this population may be at increased risk from cybersecurity threats (Grimes et al., 2007, 2010; Age-UK, 2015a; Age-Uk, 2018).

Retired, older adults are likely to be more susceptible to specific online threats. In a small scale qualitative study of three older adults, Olivier et al. (2015) suggested that they may be at particular risk of mass marketing fraud due to their pscho-social backgrounds and pre-disposing factors such as pschological vulnerability, something which was supported in a recent review of mass marketing fraud by Shao et al. (2019). Other research has identified an increased vulnerability to: telemarketing fraud (Alves and Wilson, 2008), phishing (Cho et al., 2016; Sarno et al., 2017), pension Scams (Martin and Rice, 2013; Nicholson et al., 2019), and other such targeted attacks. Those born before 1954 are also more likely to perform fewer protection behaviours and are less confident in their own ability (Jiang et al., 2016; Nicholson et al., 2019). Furthermore, they have less trust in protective resources and are more likely to be rely on others’ assistance when compared to younger generations (Jiang et al., 2016). In addition, Forget et al. (2016) interviewed 15 participants, most of whom fell within the baby boomer age range, demonstrating that security problems often arise when there are disconnects between what users see as their computer security role, and what is expected of them by others. Investigating these potential sources of vulnerability to cyber-attack is important, as these attacks can have a range of negative consequences at individual, community, and national levels (Saini et al., 2012; Canetti et al., 2017). Additionally there are ethical implications for society as a whole, since the protection of vulnerable groups could be seen as a societal responsibility (Von Solms and Van Niekerk, 2013).

One challenge in this research space is the tendency to focus on chronological age as a defining characteristic of an individual. Researchers tend to classify users into arbitrary age groups such as young children (Guan and Huck, 2012), teenagers (Wittes et al., 2016; Rahman et al., 2017), late midlife (Salovaara et al., 2010), and older adults (Chakraborty et al., 2013; Hill et al., 2015), where chronological age determines group selection, sometimes to the detriment of other socio-economic or psychological variables. Yet age is not a reliable marker for any particular user attribute, thus observing individuals based on chronological age not only risks research ageism (Vines et al., 2015), but also risks underestimating the effect of substantive life events (Shultz and Wang, 2011) and the impact they may have on cybersecurity vulnerability. This is particularly true when we consider retirement. Using semi-structured interviews with eight purposefully selected, recently retired individuals, Pettican and Prior (2011) found that retirement was identified as a new life stage, with retirement being a time of significant re-adjustment, with changes influencing perceptions of both of health and wellbeing. The varying retirement trajectories that retiring individuals face are likely to be diverse and based on a range of factors such as preparedness, planning, and the socio-economic circumstances surrounding the retirement transition. Inevitably, the post-retirement experiences of older adults are likely to vary greatly.

A large scale systematic review by Barbosa et al. (2016) outlined 26 key areas associated with adjustment into retirement, with a range of positive and negative outcomes identified in an emerging literature base. Their review demonstrates that the majority of research regarding the retirement transition revolves around physical and psychological health; however, a number of other factors are important when considering the transition from the workplace into retirement. Existing cybersecurity literature has so far failed to address the impact of retirement as a major life transition on technology use and cybersecurity vulnerability. However, some of the factors of retirement adjustment, as seen in Barbosa et al. (2016), might logically be related to cybersecurity vulnerabilities. For example, one factor identified within their review relates to finances and how financial strength influences retirement outcomes. Although it is likely that being in a strong financial position is beneficial in general when transitioning into retirement, it is likely that such financial strength might lead to increased targeting by cyber-criminals (Oliveira et al., 2017).

Another such example relates to social integration, within the Barbosa et al. (2016) review, this factor is seen to have a mixture of findings in terms of whether it is positive or neutral in its relationship to retirement adjustment. In an increasingly technological world, those who are isolated in older age may turn to social media and technology to reduce loneliness and isolation (Nowland et al., 2018), something which may open the door to certain cyber-attacks such as romance scams (Buchanan and Whitty, 2014; Whitty, 2017) or social media based cyber-attacks (Jang-Jaccard and Nepal, 2014). It is clear that a number of the factors associated with retirement might influence technology usage and as such may contribute towards cybersecurity vulnerability in retirement; however, there is presently very little research in this area. In a 4-week qualitative study, Durrant et al. (2017) investigated technology use in six recently retired older adults. They identified that older adult’s adoption and use of internet-enabled devices had a significant presence in aiding with the transition into retirement. Although they identified some security concerns relating to this technology use, very little research has looked at how these transitions, and the changing nature of this technology use might influence vulnerability to cyber-attacks.

Henkens et al. (2017) outline three areas in need of research, linking technology use to the retirement transition: (1) the impact of technology on financial preparedness, (2) the impact of technology on facilitating a better work-life balance and thus allowing people to work longer, and (3) the way technological advancements lead to improvements in psychological adjustment. While these research areas are undoubtedly important to aiding and understanding the retirement transition, we suggest that a fourth area is important but remains unaddressed – how the retirement transition can lead to increased cybersecurity vulnerability.

In this study, we aim to explore how interaction with technology changes, in both online and offline environments, as a result of the retirement transition. Furthermore, we draw from a cybersecurity literature to demonstrate how these changes are associated with the implicit cybersecurity vulnerabilities that we see in older adult populations. In doing so, we seek to provide a foundation for future work that investigates how retirement, as a major life transition, might act as an antecedent to cybersecurity vulnerabilities in post-retirement life.

## Materials and Methods

### Participants

Face to face and online sampling methods were used to search for eligible participants. A post was placed on Facebook in January 2018, which asked directly for participants, but also requested that people snowball on the recruitment information to anyone who might be eligible to take part. Once potential participants had contacted the research team, an interview was arranged at the participant’s home. A total of 12 participants from the North East England, United Kingdom, took part in the study. Although we did not originally specify a specific number of participants prior to conducting the study, due to the difficulty in establishing such measures (Levitt et al., 2018), we ceased data collection at the point which we reached data saturation, “the point at which no new themes or information arose” (Guest et al., 2006). The number of participants involved in this study is in-line with a range of existing qualitative studies in the area of cybersecurity (Olivier et al., 2015; Durrant et al., 2017; Fujs et al., 2019). The sample consisted of seven females (aged 59–74 years) and five males (aged 53–68 years) (see Table 1) who met the criteria; that they had experience in using technology, and had retired within the past 5 years. These participants were from a diverse range of backgrounds with varying technical expertise ranging from careers in retail through to engineering.

**TABLE 1:** A descriptive overview of participants.

Participants	Age (years)	Retired length	Sex	Pre-retirement work
P1	59	4 months	Female	Worked as a nurse for most of her career before moving into a role as a manager. She is married and living with a chronic health condition.
P2	60	2.5 years	Male	Project manager who worked for BT is married to P6.
P3	68	3 years	Male	Worked as an engineer for 30 years before spending 15 years in project management at BT.
P4	60	4.5 years	Male	Worked in a ministerial role relating to schools’ funding.
P5	59	6 months	Female	Worked as a technical support operator giving IT support and building IT systems.
P6	67	2.5 years	Female	Retail shop assistant. Is married to P2.
P7	66	5 years	Female	Worked as team leader in a café on a University campus.
P8	63	2 years	Female	Worked as a nurse for most of her career but ended career being a manager to other nurses. Is married to P9.
P9	65	1 month	Male	Worked as a security engineer fitting and maintaining ATMs, is married to P8.
P10	62	18 months	Fe	Retired 4 years ago but following a 3-month break this participant returned to work as a FE school vice-principal. Retired again 18 months ago.
P11	74	3 years	Female	Worked as a GP practice manager.
P12	53	2 years	Male	Worked as a self-employed charity worker.

### Materials

An interview schedule was created based on factors identified within the Barbosa et al. (2016) systematic review of retirement adjustment. First, the 26 factors of retirement research were screened to identify which factors were likely to influence changes in technology usage or increase cybersecurity vulnerability in any way. Following this review, six major factors were identified: (i) social situation, (ii) online/technology adoption, (iii) identity transitions, (iv) psychological wellbeing/personality change, (v) support structures, and (vi) financial change. Of the 26 factors attributed to retirement adjustment, these six factors were seen not only to be related to retirement, but likely to have some influence on technology usage. Interviews began by asking the participant to outline what had happened to them during their transition from the workplace into retirement. The interview then went on to ask what the biggest changes were across their retirement transition and what impact these changes had had on their day-to-day lives. The known factors, which were likely to change as a result of the retirement transition, were included as prompts within the interview to stimulate discussion. Prompts around computer and technology use were also included to stimulate relevant discourse.

### Procedure

Ethical approval was obtained from the psychology ethics board within the University of Northumbria at Newcastle upon Tyne. Semi-structured interviews were conducted in participants’ own homes, so that their devices were present and could be used to stimulate discussion. During the interview, if a participant had mentioned a specific digital device, or if it was present in the room, they were asked if they could talk about how they used the device, and if they would show the researcher the sorts of apps or software packages that they typically used. Interviews took approximately 1 h and were structured around three main topics: (1) what participants experienced in the lead up to their retirement, (2) what the participant saw as the biggest changes to their life over this period, and the reasons behind this, and (3) if and how the participants online behaviour had changed across the retirement transition.

## Findings and Discussion

### Analysis Procedure

The data was analysed using NVivo 11 software by the first author using a “soft template” (King, 1998), where the six *a priori* themes were used as an initial coding guide in accordance with an iterative template analysis (Brooks et al., 2015). Halfway through coding, and following discussion of the data with other authors, these themes were revised, resulting in a final set of six themes described below.

### Themes

During the coding phases, it became clear that emerging themes generally related to areas of *loss* rather than change, and thus the initial template was revised to reflect this. Losses were typically accompanied by compensatory behaviours, which, in some cases, contributed towards cybersecurity vulnerabilities in older adults. Each area of loss also carried with it emotional implications, which may have been the driving force behind attempts to remedy these losses. Each of these “losses” is discussed below, followed by an explicit articulation of the cybersecurity implications of that loss.

### Changes in Social Interaction

Upon leaving the workplace, an individual’s social infrastructure changes and in most cases, the social and emotional support from workplace colleagues is lost. Our participants had typically made the transition from working full time (around 37.5 h per week) to being fully retired and this drop-off in working hours led to rapid social change. For some, the loss of colleague interaction occurred immediately, as they had chosen not to maintain contact with colleagues. Others described their attempts to keep in touch with colleagues, although this too gradually deteriorated over a period of time.

*P6: I did socialise with people from work. Not a lot, but once I had retired, that got less and less, and it was sort of once a month I would speak to the girls from work, then it sort of got to once every couple of months and people kept in touch with me once I retired, but then as the months went on it got less and less and now*…*well two and a half years now since I retired, I virtually don’t see anybody from work at all.*

Nearly all participants described a vacuum in their social infrastructure. For many, social interaction had revolved almost entirely around work colleagues, and this meant that rebuilding social interaction post-retirement was difficult.

P1: A lot of nursey people do hang about with nurses, so when you stop doing that you find that trying to spread your group of friends a bit wider is a bit tricky.

This loss led to increased feelings of isolation and loneliness in many participants. This was especially apparent when the loss of social interaction felt like a slow process of neglect.

P6: When you are at work there is lots going on “oh we’re planning a night out on Friday, are you going to come?” Once you are out of the picture I think you are soon forgotten.

Generally, social loss was seen as highly negative. This finding is not surprising and is entirely consistent with the observations of Kloep and Hendry (2006), who demonstrated that people who become attached to colleagues are unhappy at losing them as part of their social infrastructure. Dorfman (1992) argued that the loss of colleague interaction was rated as the most negative aspect of retirement. Nahum-Shani and Bamberger (2009) found that in those with a large number of working hours, retirement not only led to a loss of colleagues, but also led to a decrease in emotional support overall, i.e., work friends who were previously strong emotional support structures were no longer available to the retired individual. It may be that people look to refill this social loss not only for interaction, but also for the emotional support it provided.

#### Renewing Social Interaction

In compensation, participants sought out new social opportunities via taking on new hobbies, joining groups, volunteering, and providing support for the family.

P5: One of the purposes behind me deciding to do some volunteering, I picked the library specifically so that I could meet people in the Low Fell area, because I have never had children, I have never done the school gate business, I don’t really know anyone, apart from immediate neighbours.

For those who were married, a renegotiation of the marital relationship was required to establish whether this loss of colleague interaction would be replaced by more time spent together.

P8: Now he is retired, we are kind of like sorting out how we can get on with life as two people again, instead of one working and one not working.

They described turning to technology to facilitate social interaction with those outside of their immediate home. Some started WhatsApp messaging; others joined online social networks as a means to meet new people.

*P8:*…*That has definitely increased since I have retired, the texting and the emailing and the WhatsApp.*

*P4:*…*Because the children are growing up there is a lot more sending photos, because my nephews and nieces, most of them have got kids now, so again its photos of the kids, a LOT more texting, a lot more texting actually because I have a lot more time to do it.*

Some participants described the way their social interaction now revolved around family life. They explained how their family members had bought them devices or encouraged them to use online social networks.

*P8: She gave me this phone so that I could receive photographs and so that she could Skype me. Not my Skype her, but her Skype me. And*…*FaceTime? Is it FaceTime? So that kind of thing.*

In line with these findings; Peek et al. (2016) found that it was common for families to buy devices for their older relatives and in general those within the individuals social support structure facilitated the use of technology. However, within our sample, not everyone felt competent or confident in the use of such devices and some reported that there were emotional implications in using these devices such as general fear and anxiety around unfamiliar device usage.

#### Vulnerabilities Arising From the Loss of Social Interaction

As retirees seek to build a new social life, they may turn to technology and social networking platforms to build up communication with family, find new people with common interests or new ways to express themselves (Tosun, 2012). However, online social networks are recognised as one of the biggest emerging threats to cybersecurity and privacy (Jang-Jaccard and Nepal, 2014). Increasingly, these outlets are being used to spread malware, gain information for identity theft or to seed romance scams. As retirees take to online social networks, they may increase their vulnerability to attack, particularly if they are not competent or confident with the technologies they are using. Retirees must ensure that they have anti-malware up to date and active if using such sites as they become prone to phishing attacks, romance scams, and grandparent attacks (Alves and Wilson, 2008; Age-UK, 2015b).

### Changes in Finances

Most participants experienced an immediate loss in income upon leaving the workplace. Some were financially prepared, meaning that their salary was substituted by a good pension, and reduced outgoings, e.g., being mortgage free. Finances varied among the participants with a few reporting that they were financially better off overall since retiring. More commonly, however, people experienced a large loss in their income, which resulted in changes to their financial behaviour and attitudes.

P11: One of the biggest transitions and the worst part for me is the lack of money. Um, suddenly going down from having a salary to a pension that worked out to be much less than I believed I would have received.

Participants had to change their lifestyle in order to live within their means. Participants reported managing their spending more carefully; being careful not to overspend and ensuring they sought value for money.

P5: Oh god yeah, my pension is about a 1/3 of what I used to get paid and although I have a decent amount of savings, I am finding it very interesting having to actually watch what I spend on a month by month basis.

P8: I am more careful with my money because I don’t have the disposable income I used to have, and it’s fun in a way hunting for a bargain and when you go out you get concessions because of our age. It just makes you look at money in a different way.

Financial loss had an impact on multiple aspects of life and had consequences for other retirement related losses. For example, the need to limit expenditure further, amplified the social interaction losses as social events and club memberships were perceived as too expensive.

*P7: I definitely don’t socialise like I used to, because I was out every week, every single week, I was out every weekend. Q: Why don’t you do that anymore?*…*I think of the money, do you know what I mean? Because when I worked [*…*] you would probably spend £70 on a night out and that is a lot of money when you are on your pension, that is nearly your week’s shopping.*

P11: Until I left work I was a member of xxxx cricket club, but I can’t afford that any longer, so I always went to matches as much as I could at xxxx, but I don’t do that anymore, I just can’t afford it.

For those without a car, reliance on public transport (often perceived as inaccessible or inflexible) led to further forms of isolation.

P11: It means I can’t afford to run a car any longer so that is a big change. I have had a car for many, many years um, certainly since my late 20s I’ve always had a car that I’ve run, even when I was really hard up, then it was still easier to do it than it would be now, so yeah that’s one of the real big disadvantages.

This resonates with the findings of Davey (2007), who reported a range of negative outcomes associated with the loss of a car, including difficulties in carrying out day-to-day tasks, going to see friends or shopping without assistance. These findings are also highlighted in Luiu et al. (2017); their review of the literature surrounding the implications of losing access to personal transport in older age.

Some participants were understandably frightened by loss of income.

P9: So that has gone down just to my two pensions. It’s a bit – that’s what frightens you at first and you think bloody hell, where is it, before I had the money if I wanted to go for a day out.

Post-retirement satisfaction and happiness have both been found to relate to financial status (Choi, 2001; Kim et al., 2001; van Solinge and Henkens, 2008). Burr et al. (2011) found a strong association, in that a good, stable income led to positive affect whereas poor financial status led to negative affect.

#### Vulnerabilities Arising From Financial Changes

Participants who were financially comfortable, post-retirement, reported very little in the way of associated behaviour change. However, those who had experienced financial loss, reported being much more attentive towards finances, with a number of new reported behaviours, including greater interest in online banking as a means to manage finances well:

P4: Online banking is something I have always done but I am much tighter on, but before I retired, and I didn’t really need to worry much there was always kind of enough money for what I wanted, now I have to be very careful.

This suggests that financial loss during retirement can be protective in a cybersecurity context, as the individuals attention becomes focussed on protecting their limited resources. This is supported by existing literature which indicates that those with a higher income are less risk and loss averse (Hjorth and Fosgerau, 2009; Sheehy-skeffington and Rea, 2017). Grable (2000) also found that those with a higher income and higher education level had a greater risk-taking propensity.

However, while those with stretched finances may engage in more protective checking behaviours, financial loss could lead to other cyber-vulnerabilities, e.g., through using second-hand technology, something which is especially problematic if such behaviours are not perceived to be risky at the time. One participant (P4) reported how he experienced a large financial loss following retirement, and had bought an old used laptop for £80 as well as purchasing anti-virus software from local paid IT help. He described this as his “clunky laptop.” It had an outdated operating system but was his main portal for accessing information, exchanging emails and downloading information from the Internet.

Financial loss could, thus, lead to unsafe behaviours such as purchasing of used, outdated or inherently unsafe devices. Some who are struggling financially may rely on hand-me-down devices from friends or relatives in an attempt to avoid spending precious financial resources on new technology. A lack of financial stability may also hinder people from buying security software, paying for IT help when required, and relying on those available to the individual (Dimond et al., 2010; Nicholson et al., 2019) regardless of their ability to provide good technical support.

### Loss of Sense of Purpose

The workplace can provide people with a sense of purpose or strong professional identity. Participants described feelings of loss around their former role, with some saying they no longer felt that they had a place in society, while others described feelings of guilt at no longer being in useful employment.

Some participants had worked in specific job roles for their entire working lives and their working role had become a large part of their self-identity. Upon retirement, they were forced to re-assess their identity, and this could be difficult.

*P1: It does kind of dominate your life, it sounds pathetic really, you are even a nurse when you are not at work, and you know. [*…*] It’s not being a nurse anymore, I find that quite odd.*

*P5: I have always really defined myself in a large part by what I do, and I suppose work was always very important to me because it took a lot of my life and now I don’t do that anymore I am JUST retired*…*I am JUST*…

Conversely, retirement had relatively little identity impact for those who were unhappy in their pre-retirement roles.

*P4: I think the difference is because I didn’t like my job for the last few years, I didn’t proudly identify myself with the role, it was “this is what I am doing to earn enough money” and that’s how it felt. And so, I didn’t*…*it wasn’t like losing an element of my identity, or the element of my identity that I lost didn’t like anyway.*

Role theory is a transitional theory that relates to specific roles gained and lost across the life course and may be particularly helpful when investigating the loss of a sense of purpose in recent retirees (Wang et al., 2011). Retirement acts as a role-transition (Wang, 2007) which may lead to a loss in feelings of purpose. Kim and Moen (2002) outline how, from a “role-enhancement” perspective, the loss of a career leads to feelings of “role loss” which in turn drive feelings of psychological distress and loss of morale. Alternatively, leaving a role that the individual is unhappy with, can lead to a reduction in “role strain” (Kim and Moen, 2002).

The loss of a workplace role can damage one’s self-identity (Osborne, 2012) or one’s self-esteem (Bleidorn and Schwaba, 2018) although this can depend upon the way the exit from the organisation is handled (Damman et al., 2015). Our participants used emotive language when discussing role loss following retirement, using terms such as “feeling useless,” experiencing “crises,” or likening the experience to “jumping off a cliff.”

*P5: I am having a bit of a very low-key crisis of wondering where I fit in the world, but*…*I don’t think it is anything I won’t get over.*

*P6: I think the biggest change is that I felt*…*It’s difficult to describe*…*not that I was useless, but I felt like I wasn’t*…*that I didn’t have any valuable contribution to make.*

Such distress can act as an impetus to re-fill this role loss. Participants took on a variety of new roles in retirement. If they had grandchildren living nearby, they generally reported taking on more active roles as grandparents.

*P10:*…*The other thing that takes over when you retire, when you’re a grandparent, is visiting the little ones.*

Others took on roles such as volunteering, turning to part time work or increasing the amount of time spent doing hobbies and activities. One recent retiree said he did not yet know what to do with his spare time, likening the experience to an earlier life transition, that of leaving school.

P9: I’m at the point now, like just before I left school not knowing what I want to do – it’s like, when you leave – where are you gonna go? I’m sitting here scratching my head thinking I don’t know – how long that will take I don’t know.

Thoits (2012) describes the ways that taking on a new role (e.g., volunteering) can lead to increased feelings of self-worth, renewed feelings in a sense of purpose and better physical and mental health. Volunteer roles are popular post-retirement, as they are relatively easy to obtain, are likely to involve low stress, and are typically easy to exit. However, these roles may bring new challenges and vulnerabilities.

#### Vulnerabilities Arising From Loss of Sense of Purpose

Participants taking on new roles were sometimes given technology responsibilities, regardless of their actual ability. As noted earlier, this is predominantly a group of “baby boomers,” i.e., the first group of retirees to have had technology experience during their working lives. At times, this responsibility was accepted and at other times refused.

*P5: Well, I have started looking after the website which is not*…*a particularly difficult job it is on a contact management system, but I do the updates on it and*…*and I look after their Facebook page as well, and I make use of my laptop a lot more than I used to.*

P2: The art group has asked me to manage their Facebook page for them, one of my neighbours has asked me to get involved in the Elders group in Newcastle and help them with their web development and I’m afraid I have said no to all of them, I have spent 40 years in technology and I hate it.

Even for those without a strong knowledge of technology, new roles often led to an increase in technology use associated with communication.

P4: I am in constant contact with the people who run it, the chief executive if you like I am his line manager who I see once a fortnight, we exchange a LOT of texts and emails on that.

This can be problematic for those people who are given access to systems they are ill equipped to protect. A large increase in the amount of emails that an individual handles is likely to increase an individual’s exposure to email related threats such as phishing attacks. Parsons et al. (2019) suggests that those with more technology experience will outperform those with less in terms of avoiding phishing threats, but the vulnerabilities of a new “volunteer” might not be made explicit to a recruiting organisation.

### A Loss of Day-to-Day Routine

Following retirement, participants found themselves without a day-to-day routine, which was sometimes associated with feelings of guilt about having so much free time.

P6: I think the biggest change is that you are suddenly in an environment where you aren’t busy, you go to work, I went to work 5 days a week, then all of a sudden you haven’t got that.

P5: I find it still very hard to just not have something planned to do because I feel like I am wasting time, I feel a bit guilty.

Osborne (2012) describes the “choice dilemmas” that can lead to feelings of angst or anxiety in retirees. Siegenthaler and Vaughan (1998) found that retired women often reported feeling guilty about engaging in recreation during retirement. Again, these changes can lead to a change in behaviour.

*P5: I had gone from having a very structured life to suddenly having no structure and all of this spare time to do things, so I immediately set about putting structure in place, I volunteered at various things*…

Having more free time was a reason cited for participants taking up a range of activities: re-discovering previous hobbies, dedicating more time to existing hobbies and adopting new hobbies or activities. Again, for participants this was accompanied by an increased use of technology, as they now had more time to engage with digital devices.

*P5: Facebook I didn’t do when I worked I do a bit more of now, I watch more things on television and Chromecast, I have Netflix which I didn’t have when I worked*…*I just needed more time. I didn’t have time for anything like that. It really was precisely that.*

One participant outlined how boredom led to an increase in online social network participation.

P4: Oh yes, I didn’t use it [Facebook] at all, I think it is completely new since I retired, I have more time as well, sometimes I look at Facebook because I am a bit bored.

Tosun (2012) found that a common reason for Facebook use was to curb boredom and we found evidence of other similar online activities, driven by a need to fill the retirement hours.

P8: I text and WhatsApp friends as well, quite. I guess daily really, I will sort of text people and ask, how is your mum and when are we meeting up and they will WhatsApp me back and things. That has definitely increased since I have retired, the texting and the emailing and the WhatsApp. Because I have the time to do it now.

Barnett et al. (2012) outline how retirees need to replace their working day routine with new routines in retirement for the purpose of maintaining a feeling of control and a sense of purpose over their lives. Beck et al. (2010) focussed on the initiation and maintenance of physical activity in retirement, recognising the close link between a loss of routine and a loss in a sense of purpose. Ekerdt and Koss (2016) found that routines were seen as vital by retirees for a number of reasons, one of which is to address the open-endedness of retirement and to instil a sense of purpose and meaning to one’s post-retirement life.

#### Vulnerabilities Arising From Loss of Day-to-Day Routine

Having more free time in retirement almost inevitably meant that our participants spent more time using technology. Choi (2008) suggests that one’s online routines and the way in which these routines are managed, provide opportunities for victimisation in an online environment. We noted earlier that social media use is one of the biggest emerging threats for cybersecurity (Jang-Jaccard and Nepal, 2014), but boredom can also lead to increases in things like online play which brings a number of cybersecurity concerns, particularly when that play is associated with apps downloaded onto smartphones and tablets (Lu et al., 2012; Ahvanooey et al., 2017).

### Loss of Perceived Competency

Another major change that occurs following departure from the workplace is the immediate reduction in work based cognitive demands. Within this sample, participants discussed how they felt less “mentally fit” after retiring. Additionally, they reported a decline in their computer self-efficacy related to these perceptions of declining competence.

Participants described feeling cognitively “slower,” and attributed these losses to their retirement transition.

*P1: You find yourself reading the same thing over and over again and not taking anything in. I used to find that after a fortnight off [*…*] if I went back after a fortnights holidays I wouldn’t be as sharp as when I went off until I had revved back up. And of course, I haven’t revved up since September.*

*P2: I’m sure that would have taken me an hour or so if I was*…*you know, before I retired. This time, I kept making mistakes and it wouldn’t sort, or it wouldn’t go quite how I thought it would, so I must have spent 5 h doing three sheets of A4.*

It may be that time spent in the workplace acts as cognitive protection, allowing people to “flex their mental muscles” in regard to carrying out a broad range of tasks. Evidence by Finkel et al. (2009) demonstrated that pre-retirement job roles that involve highly complex work, resulted in better cognitive functioning following the retirement transition. We know that, regardless of chronological age, adults may show more rapid cognitive decline following departure from certain workplaces and that this is linked to the complexity of the work previously undertaken (Finkel et al., 2009; Meng et al., 2017). Gordon et al. (2019) also found that older adults, regardless of chronological age, could be divided into “cognitively young” and “cognitively old” individuals and that this was reflected in their technology usage, with “cognitively older” adults using fewer apps for longer periods. These declines may not necessarily be reflective of actual cognitive decline however.

There is no doubt that actual cognitive and physical decline occurs for many and often begins before the age of 60 (Salthouse, 2009) which may in turn, may be linked to problems in mastering new technologies or even in the everyday ease-of-use of existing technologies (Hauk et al., 2018). However, people also show a number of negative self-perceptions about ageing (Sargent-Cox et al., 2012; Robertson and Kenny, 2016) which in turn can lead to doubts about competence beliefs. The perceptions of declining competence that retirees report following departure from the workplace, may instead be related to declines in self-efficacy rather than actual cognitive decline. Self-esteem gradually rises across the life course, starts to decline around the age of 50–60 years, and continues to reduce into older age (Orth and Robins, 2014). Retirement; through losses of roles, purpose and perceived competence, especially in the oldest retirees, may intensify age related declines in self-efficacy beliefs. This may be particularly problematic as declines in self-efficacy have been associated with increased cybersecurity vulnerabilities.

#### Vulnerabilities Arising From Loss of Perceived Competence

Seeman et al. (1999) demonstrated that in a sample of older adults, low self-efficacy beliefs led to incorrect assumptions of performance decline, i.e., older adults saw themselves as declining, even though this was not the case objectively. Torrens-Burton et al. (2017) also demonstrated a discontinuity between perceived and actual behaviour in older adults. Although information processing scores were the same, those who perceived that they had higher levels of cognitive dysfunction believed that they had performed more poorly, indicating a lower self-efficacy belief.

In a technology context, Vaportzis et al. (2017) found that older adults (aged between 65 and 76 years) had feelings of inadequacy when comparing their computer literacy with those of their peers. Similarly, Marquié et al. (2002) supported this in the context of general computer knowledge as they demonstrated that older adults (with a mean age of 68.6 years) underestimated their computer knowledge when comparing themselves to a younger sample (with a mean age of 22.6 years). They found that older adults were both less confident and felt less knowledgeable, regardless of the fact that their scores were in line with their younger counterparts. Although older adults may be capable, a perception of low self-efficacy may be damaging nonetheless. Workman et al. (2008) suggest that an individual’s ability to cope with an online threat is partly based upon their self-efficacy, finding that those with lower self-esteem were more likely to engage in omissive behaviours around information security. Thus, lowered self-esteem may result in avoidance behaviours, rather than attempting to deal with threats directly.

These findings are important in the interpretation of our own data, as we found incidents where low perceived computer self-efficacy and the fear and anxiety around “doing the wrong thing” drove participants to seek sources of support, which may not always have been appropriate or safe.

*P8:*…*My granddaughter will point me in the right direction, if I get really stuck I will say “help, I’m stuck” because I am afraid that I might do something wrong and lose everything. And I don’t know how to get it all back, I am very naïve when it comes to things like that.*

Nicholson et al. (2019) has shown that older adults will often behave in just this way – showing reluctance to master new procedures and turning instead to close relatives or readily available others to fix things, without necessarily checking their credentials for undertaking the task at hand. Barnard et al. (2013) noted that having access to a particularly knowledgeable child or grandchild, may backfire, reinforcing feelings of incompetence when they see the third party confidently and competently handling technology. If older adults become reliant on others for cybersecurity support, especially if these sources are inappropriate, there is a clear risk in terms of cybersecurity vulnerability.

### Loss of Technical Support Structures

Many workplaces provide technical training and support to staff members and most have appropriate policies and procedures in place. Alongside formal IT support, knowledgeable colleagues provide technical information and advice through socially constructed “shadow security” networks (Kirlappos et al., 2014). These are all lost upon retirement (Dimond et al., 2010) and our participants recognised this as an issue. They described how the workplace had provided support in the form of bulletins, updates and dedicated IT staff and described their reliance upon workplace friends and colleagues for technical support.

*P2: Yeah. At work they are all very technical people, [*…*] so I would go to them. If I had a problem I could just phone a help desk at work. But if you phone a helpdesk when you are at home then it costs money doesn’t it?*

P1: Yeah. I don’t know who else I would ask actually [for IT help]. At work I could find anyone with an iPhone and say here this has happened, what do you think?

Participants also described a reliance on workplace support structures to keep them updated with cybersecurity threats and to act as reminders of safe practices.

P1: You don’t realise how much you rely on it for, there were banners going across the computer screen homepage all of the time telling you about them [threats] and to update.

Nahum-Shani and Bamberger (2009) found that working hours were positively associated with the depth of colleague instrumental support received (support with devices) but showed how this was lost upon retirement. Instead this was replaced by advice and support from those close (non-work) friends who tended to be immediately and easily accessible – findings similar to those reported for cybersecurity advice by Nicholson et al. (2019). It appears that the options for retirees become limited, and they have to rely on what would have been their second or third choices for support.

P11: My youngest daughter is probably the principal person who would have helped me, but now I’m living here, and she lives in London, or just on the outskirts of London so she isn’t around as much, whereas [granddaughter] lives down the road.

Some participants reported employing paid help for IT support, as they no longer had available IT support structures at all.

*P4: You see when you are working*… *[*…*] there are always people around to ask questions, that is one change, there aren’t anymore. I suppose that is why I take the machine to him [local paid help] every now and then to get it cleaned up*…

People may not want to admit incompetence to family members, feeling embarrassed about their inability to deal with threats (Selwyn, 2004). It is clear, however, that the choice of support structure in retirement may result in an increase in vulnerability to cyber-attack in retirement, depending on their trustworthiness and knowledge.

#### Vulnerabilities Arising From Loss of Support Structures

Nicholson et al. (2019) may help to clarify the mechanisms by which a loss of support structures in older adults may lead to cybersecurity vulnerability. They posit a framework in which cybersecurity information is a result of an interplay between cyber-literacy and resource availability. For a retired individual, the legacy knowledge they acquired in the workplace is often used to guide their cybersecurity behaviour, but as this information becomes more dated, they turn to other resources for support and the acquisition of new knowledge and skills. Yet as we’ve seen, the post-retirement resource landscape is very variable. Some people have a wide and knowledgeable social network. Others, with more financial stability, have bought new devices and therefore have ready access to professional IT support. This pattern has been noted by Barnard et al. (2013), who notes that retirees place themselves “at risk of being left behind” which sometimes leads them to make risky decisions or rely on outdated or inappropriate advice. We see this pattern in our own data. For example, when asked about what to do if no support was immediately available, one participant explained how she might engage in behaviour outside of her comfort zone to achieve her end-goal.

*P1: If I was confident about the website. So, if it was it was iTunes. Like the computer died [*…*] and iTunes had disappeared. I downloaded that again but with clammy hands because it had to be updated, and I am a heart in the mouth kind of IT person really.*

Surprisingly, there is relatively little in the research literature about how such challenges, and more specifically about how changes in post-retirement support structures can leave people open to attack.

## Overall Discussion

We have documented a number of losses associated with retirement and shown how these can make older adults more vulnerable to cyber-attacks. Our evidence supports the notion that retirement acts as a major life disruption and one which leads people to seek out a “new normal” (Massimi et al., 2012), i.e., a new lifestyle in which previous technological and social infrastructures are lost and are subsequently replaced with tenuous new structures that can sometimes lead to additional cyber-vulnerabilities.

Here we have tried to show the social, economic and competence losses triggered upon retirement can interact in the construction of a “new normal.” For some well-resourced older adults, with good social networks, financial stability, and a range of post-retirement interests, the vulnerabilities are not so much tied to a paucity of resources, but may be associated with taking up new challenges. The retired doctor who lives alone and downloads the best-selling apps on a new smartphone has a different risk profile to the retired sales clerk who lives in close proximity to children and grandchildren and who is reliant on their second-hand devices and background knowledge. It is unfortunate that we don’t fully recognise the different technology pathways possible following retirement, the way that these vary between individual, and the associated cyber-risks.

In terms of the implications of this work, particularly for policy and lifelong learning, we would argue the following. Firstly, we recognise that the workplace legacy knowledge for individuals will vary enormously. For those in manual labour, for example, the technology skills they possess upon retirement are unlikely to derive from workplace experience. But for those who do use technology in work, one policy recommendation we could make is to consider the extent to which, as a retirement offer, they could be given access to appropriate technical and cybersecurity expertise. On the approach to retirement, cybersecurity training packages could accompany existing retirement planning packages that are offered by some organisations. Naturally, this relies on the production of an effective cybersecurity training package that teaches the individual safe practices and where to find appropriate information. This provides challenges not only for policy makers, but also for researchers attempting to implement cybersecurity interventions targeted at older adults.

Secondly, additional support should be provided for those currently in retirement, provided in an accessible format way that empowers older adults to act safely online and promotes efficacy in engaging in safety behaviours. This is likely to begin with the promotion of government backed websites such as “Cyber Aware” in the United Kingdom, but should extend to provide an age appropriate source of information, which considers those with poorer computer literacy.

Finally, to addresses losses in day-to-day routine, social interaction, and feelings of sense of purpose, which may inadvertently lead to increased vulnerability, support should be provided to promote social groups for older adults that are empowered to provide cyber support, advice and guidance as well as provide a forum for support in which older adults can support each other. In this regard, recent work on the role of CyberGuardians within a support network is interesting.

### Limitations and Future Work

We have discussed post-retirement losses without fully considering the interactions between these, noting that the interplay between these factors may intensify their effect on cybersecurity vulnerability. For example, an individual with limited financial and social resources may have to fall back on their own legacy knowledge – but what if they previously worked in a non-technical role with limited access to training? How does such an individual understand where to go to access good quality advice and support? Understanding the interplay of retirement factors is important in knowing how to target resources to support older adults.

In addition, we should consider more closely the way that cyber-attacks map onto the retirement transition. Oliveira et al. (2017) found that older adults are at particular risk of cyber-attacks associated with health, finances, and legal ideologies. Furthermore, attacks which involved reciprocation (an award was given and the email asked for recompense in the form of positive feedback) and social proofing (the incentive to join a holiday club with other similar adults) led to a significantly greater frequency of phishing link clicks. It is likely that retirees are particularly vulnerable to targeted attacks in domains that relate to their own particular retirement losses. For example, an individual in financial difficulties may be more likely to fall foul of financial phishing emails, and an individual who has lost a social network may be more likely to fall for holiday or romance scams that promise interaction with similar others. Preparing those approaching the retirement transition for the challenges they are likely to face and the associated threats may provide an interesting avenue for future cybersecurity interventions.

Finally, we have made a case for understanding more about heterogeneity in retirement, but we have done so on the basis of a study in which our sample of participants is not properly representative of our wider society. None of our participants were drawn from Black, Asian, Minority Ethnic (BAME) groups, none had declared disabilities. They were in relatively good health, many were homeowners and most were married or with partners. All of these factors are influential – but to take the last point as an example: the marital relationship influences *inter alia* post-retirement wellbeing (Szinovacz and Davey, 2003), leisure satisfaction (Losier et al., 1993), and decision as to when to retire (Smith and Moen, 1998). Additionally, it has also been implicated in specific cybersecurity risks such as an increased risk in consumer fraud victimisation in single older adults (Lee and Soberon-Ferrer, 1997).

Other issues to consider on this same issue of heterogeneity are type of retirement (phased or full) as this can influence retirement outcomes (de Vaus et al., 2007) and the time lapsed since retirement. There has been some debate around the duration of time it takes to “transition” fully into retirement (Reitzes and Mutran, 2004), but there is no doubt that the experiences given by retirees are likely to vary depending on the time since retiring. Recruitment criteria for this study required the participant to be within 5 years of their retirement and the main reason for this was to ensure that participant could remember the experiences of their retirement transition. Future research, especially any employing larger-scale quantitative methodology, might seek a broader age range of participants to pinpoint more of the socio-demographic issues but also to determine how the impact of type of retirement, former work type, and “drift” from the workplace can predict cybersecurity risk, behaviours, and/or attitudes.

## Conclusion

This study sought to investigate how the retirement transition might lead to increased cyber-vulnerability in older adulthood. Through the use of one to one qualitative interviews with recently retired United Kingdom based older adults, we found that losses in social support structures, financial stability, and perceptions of declining competence can lead to changes in the way that technology is perceived and used. The changes to a retiree’s technological landscape, in terms of both personal and external resources are likely to increase vulnerability to cyber-threats.

## Author’s Note

This project contributes towards, but is not funded by, the EPSRC funded CSALSA (CyberSecurity Across the Life SpAn) Project in collaboration with Bath University, Portsmouth University, and Cranfield University (Grant Number: EP/P011454/1).

## Data Availability Statement

The datasets generated for this study will not be made publicly available as no quantitative data was collected, and the manuscripts may release person-identifiable information. Requests to access the datasets should be directed to the corresponding author.

## Ethics Statement

The studies involving human participants were reviewed and approved by the Northumbria University, Department of Psychology Ethics Committee. The patients/participants provided their written informed consent to participate in this study.

## Author Contributions

All authors have made substantial contributions to the conception or design of the work, and the acquisition, analysis, or interpretation of data for the work.

## Conflict of Interest

The authors declare that the research was conducted in the absence of any commercial or financial relationships that could be construed as a potential conflict of interest.
