# Corrigendum: Technological Change in the Retirement Transition and the Implications for Cybersecurity Vulnerability in Older Adults

**DOI:** 10.3389/fpsyg.2020.01306

**Published:** 2020-06-24

**Authors:** Benjamin A. Morrison, Lynne Coventry, Pam Briggs

**Affiliations:** Psychology and Communication Technology Lab, Department of Psychology, Faculty of Health and Life Sciences, Northumbria University, Newcastle upon Tyne, United Kingdom

**Keywords:** retirement transition, cybersecurity, older adults, ageing, HCI

In the original article, we neglected to include a funder. This work was also supported by the Engineering and Physical Sciences Research Council [grant number EP/P011454/1].

The authors apologize for this error and state that this does not change the scientific conclusions of the article in any way. The original article has been updated.

